# The developmental course of mental health problems among children and adolescents. Results of the KiGGS cohort

**DOI:** 10.17886/RKI-GBE-2018-028

**Published:** 2018-03-15

**Authors:** Franz Baumgarten, Kathrin Klipker, Kristin Göbel, Silke Janitza, Heike Hölling

**Affiliations:** Robert Koch Institute, Berlin, Department of Epidemiology and Health Monitoring

**Keywords:** MENTAL HEALTH, MENTAL DISORDERS, PHASES OF LIFE, CHILDHOOD AND ADOLESCENCE, HEALTH MONITORING

## Background

Children and adolescents with poor mental health are more adversely affected in their quality of life than those with physical illnesses [1]. Therefore, an analysis of the factors that contribute to the development of mental health problems during the course of a child’s life can specifically be used to develop appropriate interventions and reduce the psychological strain faced by young people. Mental health problems among children and adolescents are indicated when their behaviours and feelings do not comply with social expectations given their age as well as their stage of development [2]. In accordance with this definition, 20% of children and adolescents show signs of mental health problems in Germany. The prevalence of poor mental health remained stable over two survey periods of the German Health Interview and Examination Survey for children and adolescents (KiGGS, 2003-2006 and 2009-2012) [3]. However, the development of emotional and behavioural problems identified among Children and Adolescents compared to adults is characterized by permanent changes. Mental health problems disappear within one year in every second child. Nevertheless, symptoms can persist over a long period of time and may increase over the course of a child’s development [4]. In order to identify risk groups and phases of life during which young people are most vulnerable to mental health problems, longitudinal data from the KiGGS cohort were analysed according to gender and age.

## Indicator and methodology

Data on mental health problems were collected using Goodman’s [5] Strengths and Difficulties Questionnaire (SDQ), a validated, internationally accepted instrument. In accordance with the purpose of the KiGGS study, the questionnaire was used to identify risk groups for mental health disorders in children and adolescents among various population groups. The screening questionnaire included subscales for emotional symptoms, peer relationship problems, conduct problems and hyperactivity/inattention. All analyses were based exclusively on the parent-rated SDQ of 3 to 17 year-old participants. A total of 6,459 participants (3,198 girls, 3,261 boys) were surveyed at the KiGGS baseline study (2003-2006) and KiGGS Wave 1 (2009-2012). In order to examine the course of mental health problems among children and adolescents at various stages of their lives, different age groups were analysed (32.2% were aged between 3 and 5 years, 35.1% were aged between 6 and 8 years, and 32.8% were aged between 9 and 11 years at the KiGGS baseline study). The analysis focused on children and adolescents with normal and abnormal mental health problem scores at the KiGGS baseline study, who displayed abnormal scores at KiGGS Wave 1. Probabilities were calculated for all transitions between statuses of mental health problems (i.e. normal to abnormal or abnormal to abnormal) at the KiGGS baseline study and Wave 1. The possibility of selective (re)participation was partially corrected by multivariate weighting [6].

**The KiGGS study**

The German Health Interview and Examination Survey for Children and Adolescents

**Data owner:** Robert Koch Institute

**Aim:** Providing reliable information on health status, health-related behaviour, living conditions, protective and risk factors, and health care among children, adolescents and young adults living in Germany, with the possibility of trend and longitudinal analyses

**Study design**: Combined cross-sectional and cohort study

**KiGGS survey waves**

► KiGGS baseline study (2003-2006), examination and interview survey
► KiGGS Wave 1 (2009-2012), interview survey
► KiGGS Wave 2 (2014-2017), examination and interview survey

**KiGGS cross-sectional study**

**Population:** Children and adolescents with permanent residence in Germany

**Age range:** 0-17 years

**KiGGS cohort study**

**Sampling:** Re-invitation of everyone who took part in the KiGGS baseline study (n=17,641) and who was willing to participate in a follow-up

**Age range KiGGS Wave 1:** 6-24 years (n=11,992)

**Age range KiGGS Wave 2:** 10-31 years (n=10,853)

More information is available at www.kiggs-studie.de/english

## Results

**Figure 1:**
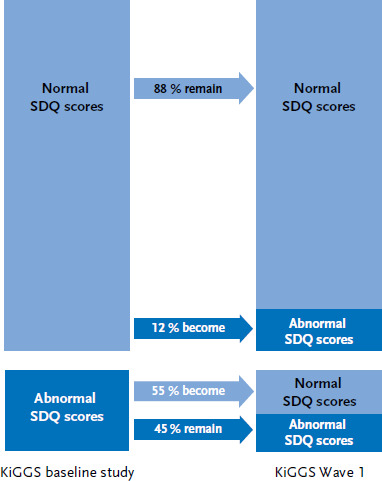
The course of individual mental health problems (according to SDQ scores) among children and adolescents at the KiGGS baseline study and KiGGS Wave 1 (n=6,459) Source: KiGGS baseline study (2003-2006), KiGGS Wave 1 (2009-2012)

Among children and adolescents with no mental health problems at the KiGGS baseline study, 12% displayed emotional and behavioural problems at KiGGS Wave 1; while 88% had no mental health problems at both survey periods (Figure 1). Only every second child and adolescent who had mental health problems at the KiGGS baseline study still displayed symptoms at KiGGS Wave 1.

**Figure 2:**
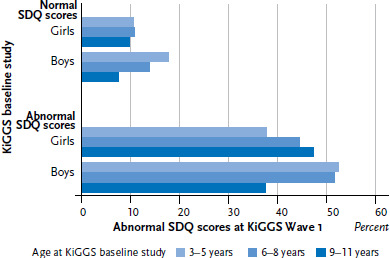
The presence of mental health problems (according to SDQ scores) at KiGGS Wave 1 for children and adolescents with normal and abnormal mental health scores at the KiGGS baseline study according to gender and age (n= 6,459) Source: KiGGS baseline study (2003-2006), KiGGS Wave 1 (2009-2012)

Differences in the course of mental health problems among boys and girls were identified with respect to different age groups (Figure 2). The proportion of children and adolescents who showed no symptoms during the first survey period (KiGGS baseline study) but displayed abnormal mental health scores six years later (KiGGS Wave 1) was highest (18%) among 3 to 5 year-old boys (compared to all other age groups and girls). Among boys, this proportion decreases with age, dropping to 8% among 9 to 11 year-olds, whereas the proportion of girls affected by mental health problems remains relatively constant across age groups.

The proportion of children and adolescents who showed mental health problems during the first survey period (KiGGS baseline study) with persistent abnormal scores at KiGGS Wave 1 was highest (52%) among 3 to 5 year-old boys (compared to all other age groups and to girls). The proportion of boys who demonstrated abnormal symptoms during both study periods decreased to 38% among children aged between 9 and 11 years. Meanwhile, girls with abnormal scores at both study periods showed an increase with age (38% vs. 45% vs. 47%).

## Discussion

Our results emphasise that a large proportion of children and adolescents showed no mental health problems (i.e. had normal scores) at both study periods. Overall boys were more likely to display emotional and behavioural problems compared to girls [3]. The occurrence of symptoms screened for by the SDQ undergoes permanent changes during the developmental process. Descriptively, boys at pre-school age (3 to 5 year-olds) and the end of primary school (9 to 11 year-olds) are most vulnerable to the onset of mental health problems. In addition, boys not only develop more problems during this stage of life compared to girls, moreover, the symptoms they do develop are also more persistent. The proportion of boys with emerging and persistent mental health problems reduces with age. Compared to boys, the proportion of girls with emerging mental health problems remains constant until adolescence. However, the proportion of girls who show persistent symptoms increases over both survey periods with advancing age. Girls seem to be particularly vulnerable to mental health problems during the transition from the end of primary school (9 to 11 year-olds) to late adolescence (15 to 17 year-olds). Compared to boys, emotional and behavioural problems are more persistent during this period in descriptive analysis.

The differences in the individual courses according to gender and age can be partly explained by categorising mental health problems into inward (internalising) and outward (externalising) symptoms [7]. Externalising mental health problems (i.e. aggression and inattention) tend to be reported more frequently among boys, internalising problems (i.e. anxiety and depression) are more common among girls [3, 8]. In general, externalising mental health problems are transient over the course of childhood and adolescence, which may be an explanation for the decrease in these symptoms among boys. Among girls, mental health problems increase over time, as internalising symptoms become more pronounced with age [8]. Still, the reported values are probably underestimated because internalising mental health problems are comparatively difficult to detect by parents and, therefore, less frequently identified [3].

Future analyses will need to particularly focus on the significance of psychosocial changes in the transition from childhood to adolescence and early adulthood (such as separations from parents, the importance and influence of friends and associated risk-related behaviour) on the course and stability of mental health problems. Thus, it is possible that a highly vulnerable child may not develop mental health problems until adolescence, because they might have been compensated in an earlier, psychosocial more stable developmental stage. In addition, psychosocial protective factors might help children develop into healthy adults despite displaying mental health problems during childhood. These and other questions could be addressed using data from the next wave of the KiGGS longitudinal survey (KiGGS Wave 2, 2014-2017) and following future KiGGS cohort studies.
